# Circular RNA expression landscapes in myelodysplastic neoplasms: Associations with mutational signatures and disease progression

**DOI:** 10.1002/1878-0261.70208

**Published:** 2026-02-19

**Authors:** Eileen Wedge, Morten Tulstrup, Gabriele Todisco, Pedro Moura, Christophe Côme, Jakob Werner Hansen, Jakob Schmidt Jespersen, Balthasar Schlotmann, Maria Creignou, Mette Dahl, Claudia Schöllkopf, Klas Raaschou‐Jensen, Krister Wennerberg, Bo Porse, Joachim Weischenfeldt, Eva Hellström‐Lindberg, Lasse Sommer Kristensen, Kirsten Grønbæk

**Affiliations:** ^1^ Department of Hematology Rigshospitalet, Copenhagen University Hospital Denmark; ^2^ Biotech Research and Innovation Center (BRIC), University of Copenhagen Denmark; ^3^ Division of Hematology, Department of Medicine Karolinska University Hospital Stockholm Sweden; ^4^ Department of Biomedical Sciences Humanitas University Milan Italy; ^5^ The Finsen Laboratory, Rigshospitalet, Copenhagen University Hospital Denmark; ^6^ Department of Hematology Odense University Hospital Denmark; ^7^ Faculty of Health and Medical Sciences, Department of Clinical Medicine University of Copenhagen Denmark; ^8^ Department of Biomedicine Aarhus University Denmark

**Keywords:** circular RNA, myelodysplastic neoplasia, non‐coding RNA, RNA sequnecing

## Abstract

Myelodysplastic neoplasms (MDS) are heterogeneous malignancies originating in hematopoietic stem cells. In this explorative study, we carried out ultra‐deep total RNA sequencing on FACS‐sorted CD34+ bone marrow cells from 71 patients and eight healthy age‐matched controls. We investigated the expression of circular RNAs (circRNAs), a group of noncoding RNAs produced by back‐splicing of nonadjacent splice sites. Key findings were further explored in an independent cohort of 118 patients with MDS and ring sideroblasts. circRNA abundance was higher in the disease groups than in controls, and different spliceosome mutations were associated with distinct circRNA expression patterns. Expression of the proliferation‐related gene *MKI67* was negatively correlated with circRNA abundance. High circRNA abundance was associated with a significantly increased risk of disease progression at 3 years. The majority of the 38 circRNAs that were significantly upregulated in MDS demonstrated highly correlated expression, and many were associated with risk of leukemic progression. Furthermore, we confirmed the specificity of circZEB1 expression to cases with *SF3B1* mutations. We conclude that aberrant circRNA expression is found in MDS and displays associations with disease characteristics and patient outcomes.

## Graphical abstract

In this explorative study, the abundance of circular RNA molecules in bone marrow stem cells was found to be elevated in patients with high‐risk myelodysplastic neoplasms, and to be associated with an increased risk of progression to acute myeloid leukemia. Circular RNA abundance displays associations with cellular proliferation, whilst expression patterns appear to be influenced by spliceosome mutations.

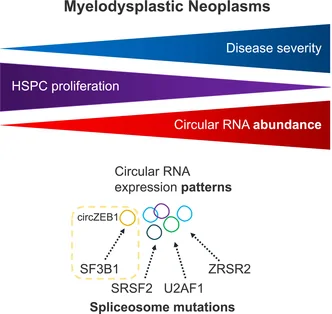

**Abbreviations**
AML: acute myeloid leukemia
CCUS: clonal cytopenia of unknown significance
CircRNA: circular RNA
CMML: chronic myelomonocytic leukemia
GO: gene ontology
HSPCs: hematopoietic stem and progenitor cells
IPSS‐M: International Prognostic Scoring System Molecular
MDS: myelodysplastic neoplasms
NGS: next‐generation sequencing
PCA: principal component analysis
RNA‐seq: RNA sequencing
RS: ring sideroblasts

## 1 Introduction

Myelodysplastic neoplasms (MDS) are cancers of the hematopoietic stem and progenitor cells (HSPCs) driven by cytogenetic alterations and somatic genetic mutations [1]. Differentiation into mature blood cells is affected, leading to cytopenias [2]. MDS may be preceded by the premalignant condition clonal cytopenia of unknown significance (CCUS) [3, 4]. Chronic myelomonocytic leukemia (CMML) is a related disease characterized by persistent monocytosis, dysplasia, and MDS‐associated mutations [3]. Progression can occur from CCUS to MDS or CMML and further to acute myeloid leukemia (AML) with a poor prognosis [3, 5, 6]. Estimating the prognosis and risk of AML progression is central to individual treatment decisions in MDS. The recently developed International Prognostic Scoring System‐Molecular (IPSS‐M) incorporates genetic mutations with the degree of cytopenia, cytogenetic abnormalities, and bone marrow blast percentage to assign risk groups. One or more genetic mutations can be detected in at least 90% of patients [7]. Recurrently mutated genes in MDS include regulators of DNA methylation (*TET2*, *DNMT3A*, *IDH2*), chromatin (*ASXL1*, *EZH2*, *STAG2*), transcription (*RUNX1*), cell cycling, apoptosis and DNA repair (*TP53*), and splicing (*SRSF2*, *SF3B1*, *U2AF1*, *ZRSR2*) [8].

There is an accumulation of genetic alterations over time, associated with disease progression [9]. MDS HSPCs display reduced proliferation [10, 11, 12], inhibition of apoptosis [2, 13], activation of innate immune pathways [14], and abnormal interaction with the surrounding bone marrow microenvironment [15]. While many pathways have been implicated, links between driver mutations and phenotypes have not been fully elucidated.

Noncoding RNAs contribute to transcriptional variation and can impact numerous cellular processes. Circular RNAs (circRNAs) are noncoding RNAs formed when a downstream 5′ splice site is joined to an upstream 3′ splice site in an event termed backsplicing [16]. We have recently shown that mutation of the spliceosome gene *U2AF1* leads to greater circRNA abundance in MDS, largely independent of changes in the expression of the linear host genes of the circRNAs [17]. Spliceosome mutations (commonly of *SF3B1*, *SRSF2*, *U2AF1*, or *ZRSR2*) are a hallmark of MDS, found in at least 50% of cases [7, 18]. We hypothesize that circRNA expression may be altered in MDS cases in association with the mutational signature and may be associated with patient outcomes.

Here, we carried out ultra‐deep total RNA sequencing (RNA‐seq) on CD34+ HSPCs from a cohort of well‐characterized patients at the time of diagnosis of MDS, CCUS, or CMML. A variety of subtypes of MDS were included, with a view to carrying out explorative analyses of the circRNA expression landscapes within this highly heterogeneous disease. In addition, circRNA expression was examined in an independent cohort consisting of patients with MDS with ring sideroblasts. Together, this work shows that circRNAs are aberrantly expressed in MDS and are associated with differences in mutational signature, disease severity, and risk of leukemic progression.

## 2 Methods

### 2.1 Study cohorts: Patient inclusion and ethical approval

The main cohort of this study included 71 patients with CCUS, MDS, or CMML from four Danish clinical sites (Table 1) and eight age‐matched (median 64 years, range 44–84) and sex‐matched (5 male, 62.5%) healthy controls, with normal peripheral blood counts. All participants provided written informed consent. The study was conducted according to the standards set by the Declaration of Helsinki and ethics approval was obtained from the National Ethics Committee of Denmark (ref. 1 705 391). The study was registered with the Danish data protection agency (ref. P‐2019‐702).

**Table 1:** Clinical and pathological patient characteristics of the mixed MDS cohort.

	MDS (*n* = 45)	CMML (*n* = 12)	CCUS (*n* = 14)
Age (median, range)	73 (43–93)	82 (55–91)	77.5 (54–88)
Sex	30 male (67%), 15 female	10 male (83%), 2 female	13 male (93%), 1 female
Disease subtype			
MDS‐LB	12	‐	NA
MDS‐IB	12		
MDS‐RS	4		
MDS‐SF3B1	11		
MDS‐del5q	3		
MDS‐biTP53	3		
CMML			
Myelodysplastic CMML		7	
Myeloproliferative		5	
Bone marrow blast %			
10–19	7	3	‐
5–9	6	3	‐
< 5	32	6	14
IPSS‐M risk group			
Very high risk	3	NA	NA
High risk	11		
Moderate high risk	5		
Moderate low risk	4		
Low risk	15		
Very low risk	7		
Cytogenetics			
Complex karyotype^a^	4	0	0
Other abnormality	14	2	2
No abnormality	25	7	11
Not performed	2	3	1
Mutated genes^b^			
*TET2*	14	9	7
*SRSF2*	11	7	4
*ASXL1*	10	4	5
*SF3B1*	11	1	1
*U2AF1*	10	1	1
*DNMT3A*	5	1	4
*IDH2*	5	1	1
*RUNX1*	6	0	1
*STAG2*	6	1	0
*TP53*	6	0	1
*ZRSR2*	3	1	3
*ETNK1*	3	0	0
*EZH2*	1	2	0
*KRAS*	0	2	1
*BCOR*	2	0	0
*CBL*	1	1	0
*NRAS*	0	2	0
*PTPN11*	1	1	0

a 3 or more abnormalities.

b Mutations detected in 2 or more patients are shown.

Patients underwent bone marrow aspirate during the initial diagnostic examination, as well as healthy controls at a single study visit. Mononuclear cells were separated using Ficoll according to standard protocols, and live cells were frozen at −130 °C until further use. Baseline clinical data including cytogenetic analysis (by G‐band karyotyping) were obtained from patient medical records. Cytogenetic analysis was missing for five patients. Follow‐up data were recorded whenever further bone marrow examinations were performed in routine clinical care, at which point further material was also collected for the study biobank. In addition, notifications of patient deaths were recorded. The median follow‐up time was 40.6 months (reverse Kaplan–Meier method).

In addition, we analyzed previously generated RNAseq data from an independent cohort of 118 MDS patients and 10 healthy controls. All patients had ≥ 5% ring sideroblasts (RS), distinguishing them from the main cohort described above. Details and methods regarding the RS cohort have been previously described [19]. In brief, CD34+ cells had been isolated by magnetic sorting, and total RNA‐seq performed on an Illumina NovaSeq6000 with read length of 150 bp and mean depth of 69 million reads (range 25–257 million). Survival data were available for the RS cohort with a median follow‐up of 47.3 months.

### 2.2 Mutation profiling

Targeted next‐generation sequencing (NGS) was performed on mononuclear cells (blood or bone marrow) for a panel of 33 genes commonly mutated in MDS (Table S1). All coding regions of these genes were included. Briefly, 100 ng of genomic DNA was used to pre‐pare libraries with the Twist Library Preparation EF Kit 1.0 (Twist Bioscience, California, USA), and libraries were enriched for the target genes using hybridization capture with a custom Twist probe panel (Twist Bioscience, California, USA). Sequencing was performed on NextSeq500 or NovaSeq 6000 instruments (Illumina, San Diego, California, USA) to obtain an average target depth of > 500×. FASTQ files were aligned with the Burrow–Wheeler aligner v. 0.7.1719 using a custom reference genome (hg38 with masked U2AF1L5 erroneous insertion). Variant callers SNVer,20 Var‐Dict21 and Mutect222 were used and the results were curated.

### 2.3 FACS cell sorting

After thawing of bone marrow mononuclear cells from patients and controls, CD45+/dim CD3− CD19− CD34+ cells were sorted using a BD Biosciences Aria III (antibody details can be found in Table S2). The purity of sorted cells was checked for 74 samples (93%); 67 (91%) were above 90%, while the remaining seven were above 70% (CD34+/CD45+). Sorted cells were frozen in DNA/RNA Shield buffer (Zymo, Irvine, CA) until RNA isolation.

### 2.4 Total RNA‐seq

RNA was isolated using the Quick‐DNA/RNA Microprep Plus isolation kit (Zymo), according to the manufacturer's instructions, including DNase I treatment. Library preparation used the SMARTer Stranded Total RNA‐Seq Kit v3 ‐ Pico Input (Takara, Kusatsu, Japan) according to the manufacturer's instructions, with 10 ng RNA input. Sequencing was performed on an Illumina NovaSeq 6000 with 150 bp paired‐end reads, at a mean depth of 280 million paired‐end reads (range 143–406 million).

### 2.5 Bioinformatics

RNA‐seq data were first prepared by removal of adapter sequences and trimming of low‐quality bases (Phred score below 20) using Trim Galore. FastQC was used to perform quality control. The filtered RNA‐seq reads were then mapped against the human genome (hg19/GRCh37) using STAR.23 Gene expression was quantified using featureCounts with gene annotations from Gencode release 37.

Reads containing back‐splicing junctions were identified using the pipelines CIRI2 [20] and find_circ [21]. To minimize false positives [22], we required detection by both pipelines in the main cohort. However, in analysis of the RS cohort, only detection by CIRI2 was required, to optimize yield in the context of lower sequencing depth, as CIRI2 is more sensitive than find_circ [23]. Normalization was performed with DESeq2 [24], with the inclusion of mRNA data for a more robust normalization by addressing both sample‐to‐sample (sequencing depth) and gene‐to‐gene effects on read counts. DESeq2 was also used for differential expression analyses and for variance stabilizing transformation prior to principal component analysis (PCA) and hierarchical clustering analyses. To quantify linear reads corresponding to each detected circRNA, we used get_flanking_spliced_reads.py (https://github.com/ncrnalab/pyutils). A pseudocount (of the lowest value in the dataset) was added to the resulting linear counts before calculating a fold change.

### 2.6 Statistical analysis

Analyses were conducted using R v4.2.0 (The R Foundation for Statistical Computing, Vienna, Austria) and graphpad prism 9 (graphpad Software, San Diego, CA). Mann–Whitney tests were used to compare expression levels between groups; two‐sided *P* values ≤ 0.05 were considered significant. Correlations were assessed using the Spearman method. For differential expression analysis, adjustment for multiple testing was carried out by DESeq2. All other *P* values were unadjusted. *PCAtools* and *ComplexHeatmap* were used for PCA and hierarchical cluster analysis, respectively, while *cmprsk* and *survival* were used for competing risks and survival analyses.

## 3 Results

### 3.1 circRNAs are highly expressed in HSPCs from patients with MDS and CCUS

Ultra‐deep total RNA‐seq was carried out on CD34+ HSPCs from 71 MDS patients and eight healthy controls. A total of 18 235 circRNAs were detected by both CIRI2 and find_circ and were present in at least two samples; these circRNAs were included for further analysis. The most abundant circRNAs accounted for the majority of circRNA reads (66% of normalized reads originated from the 1000 most abundant circRNAs in the dataset). This pattern was the same across disease groups (Fig. S1a–d). The group‐wise means across unique circRNAs were higher for CCUS than healthy controls (*P* < 0.0001), and higher for MDS than CCUS (*P* = 0.0006) (Fig. S1e). The total normalized circRNA reads per individual (circRNA abundance) was lowest in healthy controls (median 32 928), with significantly higher levels in CCUS (median 50 740) and high‐risk MDS (median 62 070) (*P* = 0.0103 and *P* = 0.0029, respectively) while circRNA abundance in low‐risk MDS and CMML (median 38 805 and 42 353, respectively) was not significantly higher than healthy controls (Fig. 1A). Similar results were obtained when considering the number of circRNAs with expression levels supported by more than five normalized reads, indicating that the increase in circRNA abundance is widespread rather than being driven by changes in a limited number of circRNAs (Fig. 1B).

**Fig. 1:**
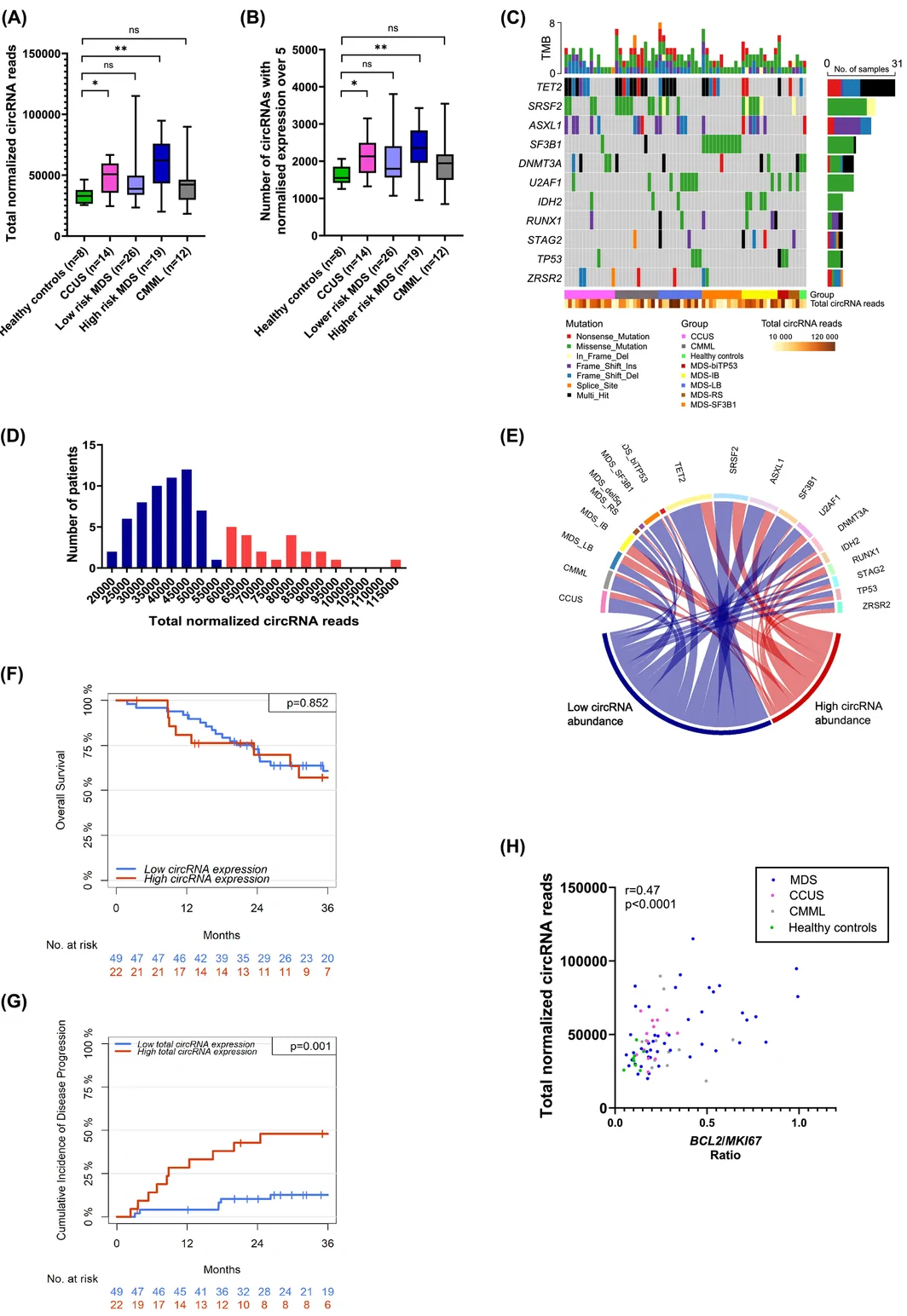
Circular RNA (circRNA) abundance and expression patterns are altered in myelodysplastic neoplasms (MDS; *n* = 45), cloncal cytopenia of uncertain significance (CCUS; *n* = 14) and chronic myelomonocytic leukemia (CMML; *n* = 12), and high circRNA abundance is associated with disease progression. (A) Total normalized circRNA reads by disease group, with MDS divided into Low Risk (International Prognostic Scoring System ‐Molecular (IPSS‐M) Very Low, Low, or Moderate Low risk groups; *n* = 26) and High Risk (IPSS‐M Moderate High, High or Very High risk groups; *n* = 19). Bars show median and quartiles, whiskers show range, comparison by Mann Whitney test (**P < 0.01, *P < 0.05, ns = not significant). (B) Number of circRNAs with mean normalized read count over 5 for each group, with MDS divided into Low Risk (IPSS‐M Very Low, Low, or Moderate Low risk groups) and High Risk (IPSS‐M Moderate High, High or Very High risk groups). Bars show median and quartiles, whiskers show range, comparison by Mann Whitney test (**P < 0.01, *P < 0.05, ns = not significant). (C) Oncoplot showing the mutations found by targeted Next Generation Sequencing, stratified by disease subtype and annotated by disease subtype and total normalized circRNA reads. TMB = tumor mutational burden i.e., total number of mutations per patient. (D) Histogram of total normalized circRNA reads, with red and blue bars indicating samples designated as high and low circRNA abundance, respectively. (E) Chord diagram showing links between disease subgroups or the presence of individual mutations and high or low total circRNA abundance (as defined in D). (F) Kaplan–Meier graph showing overall survival for patients designated as high (*n* = 22) vs. low (*n* = 49) total circRNA abundance (as defined in D). (G) Cumulative incidence of disease progression (any of CCUS to MDS or AML, MDS to AML, CMML to AML) with death as a competing risk for patients designated as high (*n* = 22) vs. low (*n* = 49) total circRNA abundance (as defined in D). (H) Total normalized circRNA reads plotted against the *BCL2*/*MKI67* ratio, analysis by Spearman's correlation (*n* = 79).

### 3.2 High total circRNA expression is associated with high‐risk characteristics and risk of disease progression

Targeted sequencing revealed a typical distribution of mutations [7, 8], with the most common being in *TET2*, *SRSF2*, *ASXL1*, *SF3B1*, and *DNMT3A* (Fig. 1C). Clonal hematopoiesis of indeterminate potential (CHIP) mutations [25] were detected in two (25%) of the age‐matched controls (one with *TET2* and *DNMT3A*, one with *DNMT3A* and *ASXL1*). Some tendencies in circRNA abundance, such as lower levels in patients with an *SF3B1* mutation, were immediately evident (Fig. 1C). Samples were grouped into high and low circRNA abundance according to the distribution of total normalized circRNA reads (Fig. 1D). Patients with high circRNA abundance (*n* = 22) were significantly more likely to have MDS with increased blasts (MDS‐IB, blast count ≥ 5%) (*P* = 0.039), as well as *IDH2* (*P* = 0.026) and *RUNX1* mutations (*P* = 0.026). Meanwhile, the low circRNA abundance (*n* = 49) group appeared enriched for *SF3B1* mutations (*P* = 0.052) (Fig. 1E). High circRNA abundance was not associated with overall survival (Fig. 1F) but did show a significant association with disease progression (CCUS‐ > MDS, MDS‐ > AML, or CMML‐ > AML) with death as a competing risk (*P* = 0.001 for difference at 3 years, Fig. 1G).

### 3.3 circRNA abundance is negatively correlated with markers of cell turnover

The circRNA dilution hypothesis [26, 27] posits that circRNAs, which are stable but inefficiently produced [21, 28, 29], become diluted in rapidly proliferating cells [26, 30, 31]. Since this model depends on cell turnover, we hypothesized that apoptosis would also influence circRNA abundance. As previous studies in MDS have reported elevated levels of the anti‐apoptotic gene *BCL2* [2, 13] along with reduced expression of the proliferation‐associated genes *PCNA* and *MKI67* [10], we compared the expression of these three genes to circRNA abundance. As expected, *PCNA* and *MKI67* were strongly correlated (*r* = 0.85, *P* < 0.0001). *PCNA* showed a weak but significant negative correlation with circRNA abundance (*r* = −0.23, *P* = 0.04, Fig. S1f), while *MKI67* showed a moderate negative correlation (*r* = −0.33, *P* = 0.003, Fig. S1g). In contrast, *BCL2* expression was moderately positively correlated with circRNA abundance (*r* = 0.36, *P* = 0.003, Fig. S1h). Given these opposing relationships, we calculated the ratio of *BCL2*/*MKI67* expression, which showed a stronger positive correlation with circRNA abundance (*r* = 0.47, *P* < 0.0001, Fig. 1H). In univariate analyses as continuous variables, *PCNA* (*P* = 0.014), *BCL2* (*P* = 0.005), and the *BCL2*/*MKI67* ratio (*P* < 0.001) were all significantly associated with risk of disease progression, while *MKI67* alone was not (*P* = 0.11).

### 3.4 Spliceosome mutations influence patterns of circRNA expression

Unsupervised hierarchical clustering using the Euclidean clustering method showed an association between high‐risk features and high circRNA abundance as already described (Fig. S1i). Euclidean clustering puts a relatively high emphasis on absolute levels, while Pearson clustering emphasizes relationships and patterns between elements by allowing for mean shifts between samples. Interestingly, Pearson clustering revealed a strong tendency for patients to cluster according to spliceosome mutation status, regardless of disease subtype (Fig. 2A). This was particularly clear for *SRSF2*, with all but one *SRSF2* ^MUT^sample clustering together. Furthermore, samples clustered according to the specific amino acid alterations of the spliceosome mutations. For example, all samples with *SF3B1* K700E mutations clustered together, except for the single case with a *ZRSR2* co‐mutation (Fig. 2B).

**Fig. 2:**
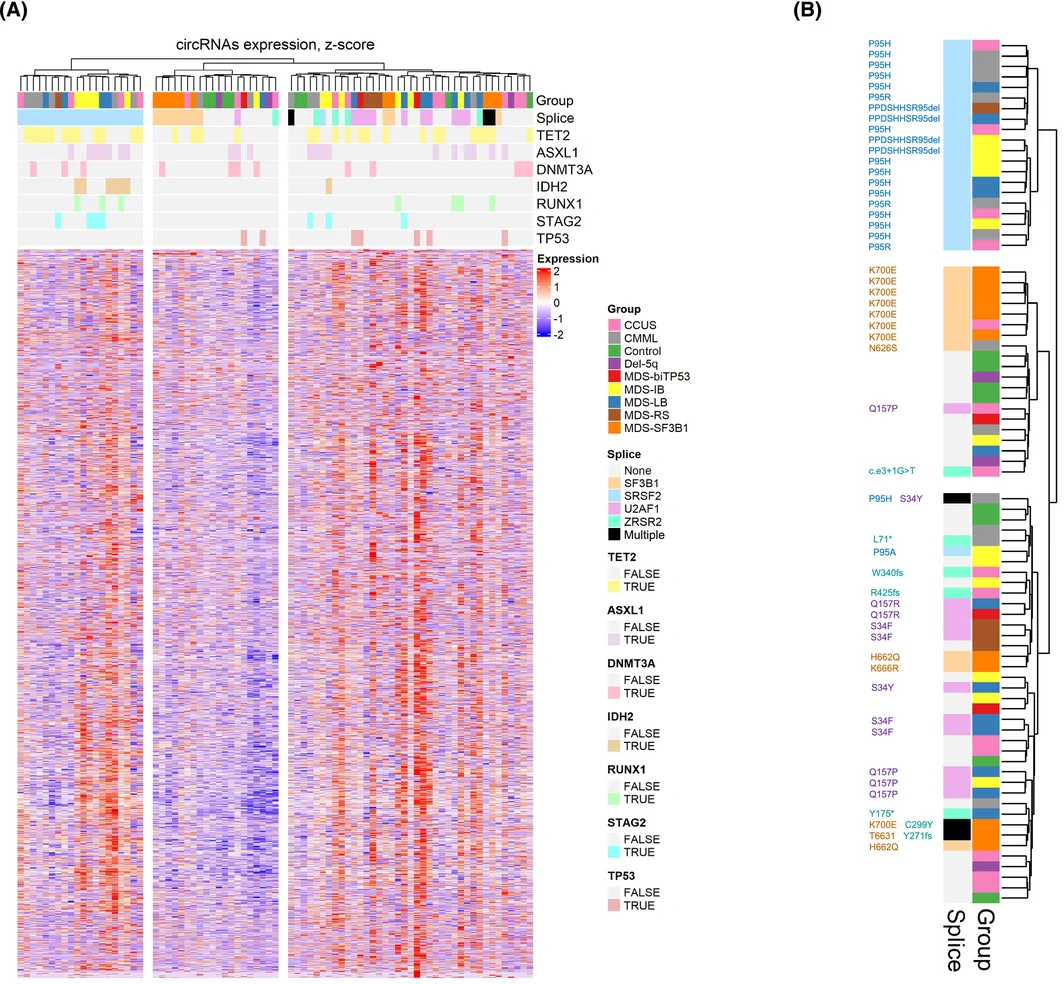
Spliceosome mutations influence circular RNA (circRNA) expression patterns. (A) Heatmap showing unsupervised hierarchical cluster analysis for the 3033 circRNAs with a normalized expression > 5 reads in one or more disease subgroup using Pearson clustering (*n* = 79). (B) Cluster annotation from panel A supplemented with the specific amino acid changes of mutations of the splicing factors *SRSF2*, *U2AF1*, *SF3B1*, or *ZRSR2*.

### 3.5 Patients with high circRNA abundance have a distinct gene expression profile associated with the innate immune system and viral response pathways

Given the apparent heterogeneity of patients with high circRNA abundance, we wondered whether high circRNA abundance could be associated with an altered gene expression profile. We compared gene expression between the 22 patients with high circRNA abundance with the remaining 49 patients. PCA of the gene expression data showed a tendency for samples with high circRNA abundance to separate from samples with low circRNA abundance on the first and/or second principal component(s) (Fig. 3A). DESeq2 analysis revealed many differentially expressed genes between the two groups (613 upregulated and 3033 downregulated, Fig. 3B). Gene ontology (GO) analysis of the upregulated genes revealed an enrichment of GO terms related to plasma membrane adhesion molecules, innate immunity and viral defense pathways (Fig. 3C,D). GO analysis of the downregulated genes showed weaker associations across a variety of biological processes (Fig. S2a,b). Interestingly, individual circRNAs whose expression correlated with innate immunity genes tended to also correlate with cell turnover markers, while other circRNAs did not display correlation with either of these groups of genes (Fig. S3).

**Fig. 3:**
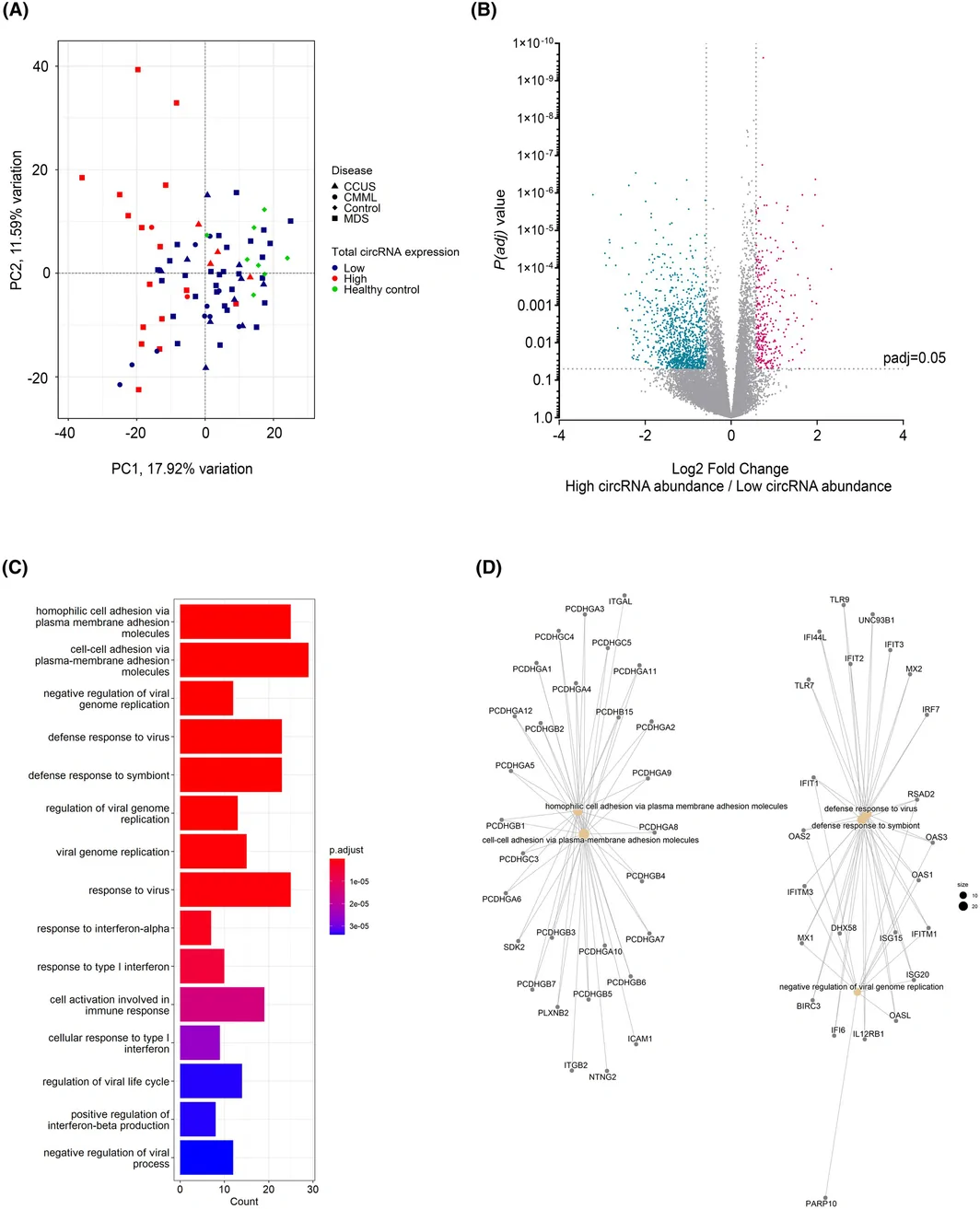
High circular RNA (circRNA) abundance is associated with changes in membrane adhesion and innate immunity related genes. (A) Principal component analysis of protein‐coding gene expression (based on the 500 genes with the greatest variance) with shapes denoting disease group and color denoting circRNA abundance group. (B) Volcano plot depicting differential expression of protein coding genes between patients with high circRNA abundance (*n* = 22) and patients with low circRNA abundance (*n* = 49), including the 3822 highest expressed genes (cutoff determined by DESeq2 independent filtering). (C) Gene Ontology enrichment analysis of genes upregulated in patients with high circRNA abundance, showing top 15 terms. (D) Cnet plot showing the upregulated genes associated with the top 5 terms from panel C.

### 3.6 circRNA upregulation is largely independent of cognate linear mRNA expression

The widespread upregulation of circRNAs was confirmed by differential expression analysis, which showed a strong skew toward upregulation in MDS relative to healthy controls, with 38 significantly upregulated circRNAs and none significantly downregulated, after correction for multiple testing (Fig. 4A, Table 2). Differences in circRNA expression were weakly but significantly correlated with changes in cognate linear expression at the same locus (*r* = 0.28, *P* < 0.0001, Fig. 4B). Overall, circRNA log2 fold changes were of greater magnitude, and the majority of differentially expressed circRNAs were associated with minimal changes in the corresponding linear expression. Of the 38 significantly upregulated circRNAs, the corresponding linear transcripts were downregulated or stable (fold change < 1.5) in 31 cases. For an additional four circRNAs, the fold change in expression was more than twice the magnitude of the fold change of the cognate linear transcript (Table 2). This leaves only three circRNAs whose upregulation may be dependent on increased host gene expression.

**Fig. 4:**
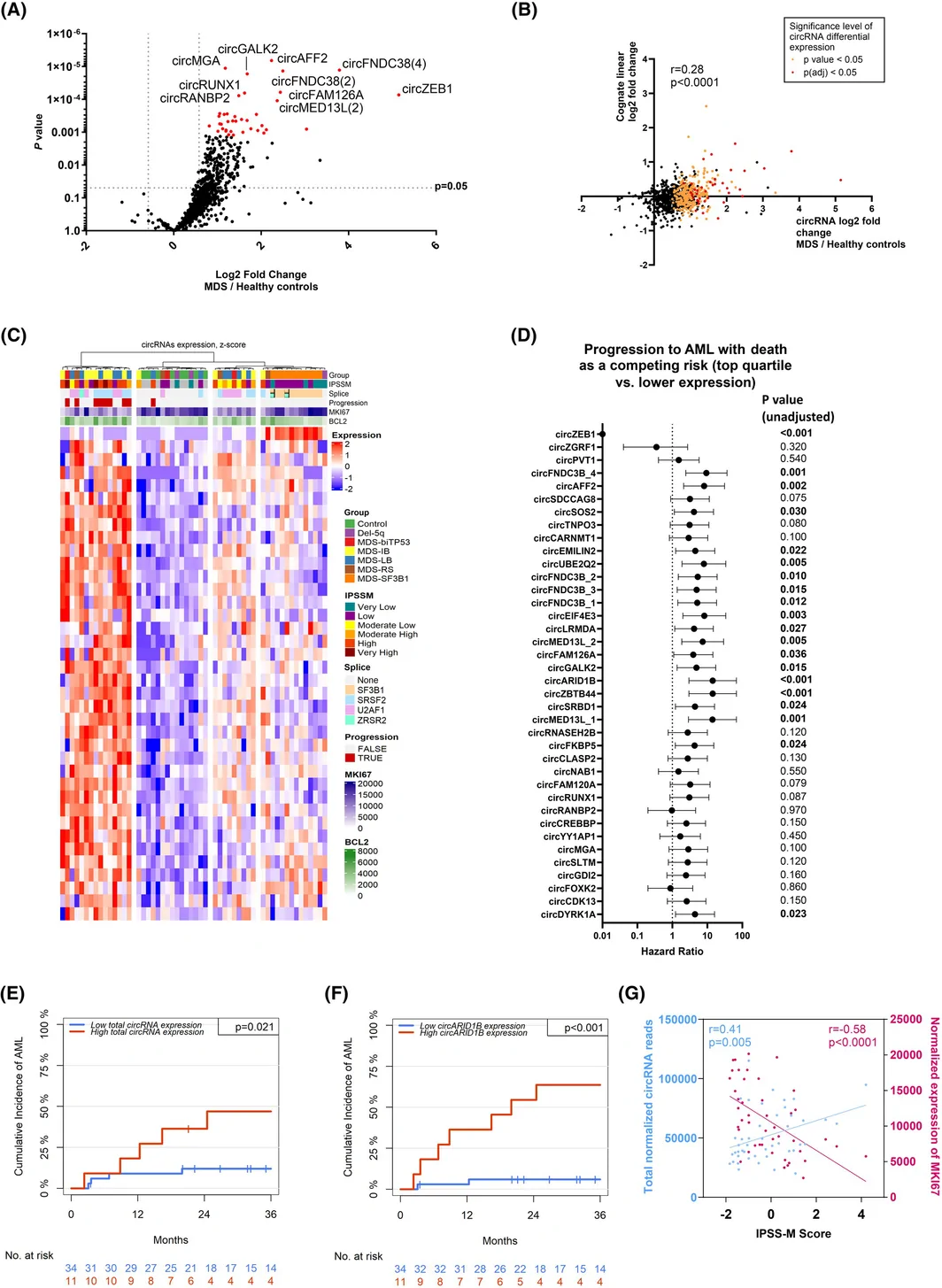
Upregulated circular RNAs (circRNAs) in myelodysplastic neoplasms (MDS) are associated with disease severity and risk of progression to acute myeloid leukemia. (A) Volcano plot depicting the differential expression of circRNAs between patients with MDS (*n* = 45) and healthy controls (*n* = 8), including 887 highly expressed circRNAs (cutoff determined by DESeq2 independent filtering). Raw *P* values are plotted, with significant *P*(adj) indicated by red dots. (B) Log_2_ fold change for circRNAs plotted against log_2_ fold change of linear mRNAs from the same locus. Orange dots indicate circRNAs, which are differentially expressed (significant unadjusted *P* value) and red dots indicate circRNAs being significant after correction for multiple testing, analyzed by Spearman's correlation. (C) Heatmap and unsupervised hierarchical cluster analysis of the 38 differentially expressed circRNAs between MDS and healthy controls. (D) Forest plot showing the results of univariate analyses for risk of progression to acute myeloid leukemia (AML) with death as a competing risk for each of the 38 differentially expressed circRNAs as continuous variables, shown in the same order as the rows of the heatmap in panel C. (E) Cumulative incidence of AML (with death as competing risk) in patients with MDS divided into the highest quartile of total normalized circRNA reads (‘High total circRNA expression’; *n* = 11) versus the remaining MDS patients (‘Low total circRNA expression’; *n* = 34). (F) Cumulative incidence of AML (with death as competing risk) in patients in the highest quartile of circARID1B expression (*n* = 11) versus the remaining MDS patients (*n* = 34). (G) International Prognostic Scoring System‐Molecular score (continuous) plotted against total normalized circRNA reads and normalized expression of *MKI67*, analyzed by Spearman's correlation (*n* = 45).

**Table 2:** Differentially expressed circRNAs (all upregulated) in MDS compared to healthy controls.

circRNA	CircBase ID	Co‐ordinates	circRNA fold change	Cognate linear fold change
circAFF2	hsa_circ_0001947	chrX:147743428–147 744 289	4.71	2.89
circMGA	hsa_circ_0000592	chr15:41988272–41 991 357	2.26	1.05
circFNDC3B_4	hsa_circ_0067990	chr3:171969049–172 016 577	13.81	2.483
circFNDC3B_2	hsa_circ_0003692	chr3:171969049–172 028 671	5.64	1.67
circGALK2	hsa_circ_0008488	chr15:49528047–49 531 564	3.20	1.28
circFAM120A_2	hsa_circ_0008951	chr7:22999874–23 030 758	5.42	1.00
circRUNX1	hsa_circ_0002360	chr21:36206706–36 231 875	3.07	1.25
circZEB1	hsa_circ_0000228	chr10:31661946–31 676 195	35.43	1.39
circRANBP2	hsa_circ_0006965	chr2:109388156–109 389 502	2.80	0.97
circMED13L_2	hsa_circ_0008151	chr12:116674625–116 675 510	5.16	1.15
circMED13L_1	hsa_circ_0000443	chr12:116668337–116 675 510	2.07	1.04
circGDI2	hsa_circ_0005379	chr10:5827104–5 842 668	2.33	0.91
circCDK13	hsa_circ_0079929	chr7:40027197–40 027 857	3.71	1.30
circTNPO3	hsa_circ_0001741	chr7:128655032–128 658 211	2.22	0.75
circNAB1	hsa_circ_0002024	chr2:191523883–191 537 878	2.05	1.29
circCLASP2	hsa_circ_0001280	chr3:33725850–33 738 425	2.63	0.90
circSDCCAG8	hsa_circ_0017241	chr1:243579003–243 589 860	2.93	1.33
circPVT1	hsa_circ_0001821	chr8:128902834–128 903 244	3.20	1.42
circFAM120A_1	hsa_circ_0001875	chr9:96233422–96 261 168	2.55	0.71
circSRBD1	hsa_circ_0005542	chr2:45773870–45 780 869	2.68	1.10
circCREBBP	hsa_circ_0007637	chr16:3900297–3 901 010	2.36	0.86
circSLTM	hsa_circ_0003713	chr15:59204761–59 205 895	2.07	1.03
circYY1AP1	hsa_circ_0003608	chr1:155644800–155 649 303	2.12	0.96
circARID1B	hsa_circ_0008519	chr6:157150360–157 222 659	4.05	1.18
circZBTB44	hsa_circ_0002484	chr11:130130750–130 131 824	1.76	1.30
circFKBP5	hsa_circ_0001599	chr6:35586872–35 610 620	3.40	0.64
circFOXK2	hsa_circ_0000816	chr17:80521229–80 526 077	2.17	1.14
circEIF4E3	hsa_circ_0001322	chr3:71733722–71 759 635	8.21	1.76
circFNDC3B_3	hsa_circ_0067991	chr3:171969049–172 025 291	4.34	1.70
circUBE2Q2	hsa_circ_0104568	chr15:76152218–76 165 909	2.29	1.12
circZGRF1	hsa_circ_0070680	chr4:113483526–113 506 881	3.79	0.89
circSOS2	hsa_circ_0007695	chr14:50616725–50 616 948	2.29	0.93
circRNASEH2B	hsa_circ_0000489	chr13:51501542–51 523 641	2.02	1.08
circDYRK1A	hsa_circ_0005955	chr21:38792600–38 845 182	2.48	1.20
circEMLIN2	hsa_circ_0004658	chr18:2890558–2 892 484	3.00	2.33
circLRMDA	.	chr10:77302261–77 307 420	4.17	1.07
circCARNMT1	hsa_circ_0004189	chr9:77631183–77 632 364	2.39	0.95
circFNDC3B_1	hsa_circ_0006156	chr3:171965322–171 969 331	2.57	2.12

A strong skew toward upregulation was also present in CCUS relative to healthy controls, along with a limited correlation with the corresponding linear expression changes (*r* = 0.26, *P* < 0.0001, Fig. S4a,b). In a comparison between MDS and CCUS, a skew toward upregulation was observed, though it was less pronounced. As before, the correlation with corresponding linear expression changes was weak (Fig. S4c,d). Finally, similar tendencies were seen in CMML relative to healthy controls (Fig. S4e,f). Given that the most pronounced changes in circRNA expression were observed in MDS, we focused on the circRNAs which were upregulated in MDS relative to healthy controls for further analyses.

### 3.7 Upregulated circRNAs are associated with an increased risk of progression to AML

The expression patterns of the 38 MDS‐associated circRNAs identified above revealed a noticeable degree of correlation, with the majority showing highest expression levels in the same cases, many of whom later progressed to AML (Fig. 4C). However, circZEB1 (hsa_circ_0000228) showed a distinct expression pattern (Fig. 4C, top row) and was highly specific to patients with *SF3B1* mutations, as has been previously reported [32]. Twenty (53%) of the circRNAs were significantly associated with risk of progression to AML (Fig. 4D). MDS‐SF3B1 is associated with a low risk of progression to AML [33]. Consequently, we repeated the analysis after excluding these patients; 13 of the 20 circRNAs remained significantly associated with progression, and five additional circRNAs gained significance (Fig. S5).

The MDS patients with the highest circRNA abundance (top quartile) had a cumulative incidence of AML of 47% (95% CI 15–74%) after three years compared to 12% (95% CI 4–26%) for MDS patients with lower circRNA abundance (*P* = 0.021, Fig. 4E). The individual circRNA, which was most strongly associated with AML risk, was circARID1B. The cumulative incidence of AML was 64% (95% CI 27–86%) in the high circARID1B expression group compared to 6% (95% CI 1–18%) in the lower expression group (*P* < 0.001, Fig. 4F).

All patients with progression to AML were in a high IPSS‐M risk group (Moderate High, High or Very High). With increasing disease severity (IPSS‐M), *MKI67* expression decreased (*r* = −0.58, *P* < 0.001) and circRNA abundance increased (*r* = 0.41, *P* = 0.005) (Fig. 4H).

### 3.8 High risk MDS is associated with increased circRNA abundance in an independent cohort

In an independent cohort of 118 patients with MDS and at least 5% ring sideroblasts (RS), we found significantly higher circRNA abundance in patients with high risk MDS compared to low risk MDS (*P* = 0.0002), while low risk cases were not significantly different from healthy controls (*n* = 10) (Fig. 5A). Consistent with the findings described above, there was a negative correlation between *MKI67* expression and circRNA abundance in MDS patients (*r* = −0.4319, *P* < 0.0001, Fig. 5B). There was no correlation between *BCL2* expression and circRNA abundance in the RS cohort (*r* = −0.03, *P* = 0.782).

**Fig. 5:**
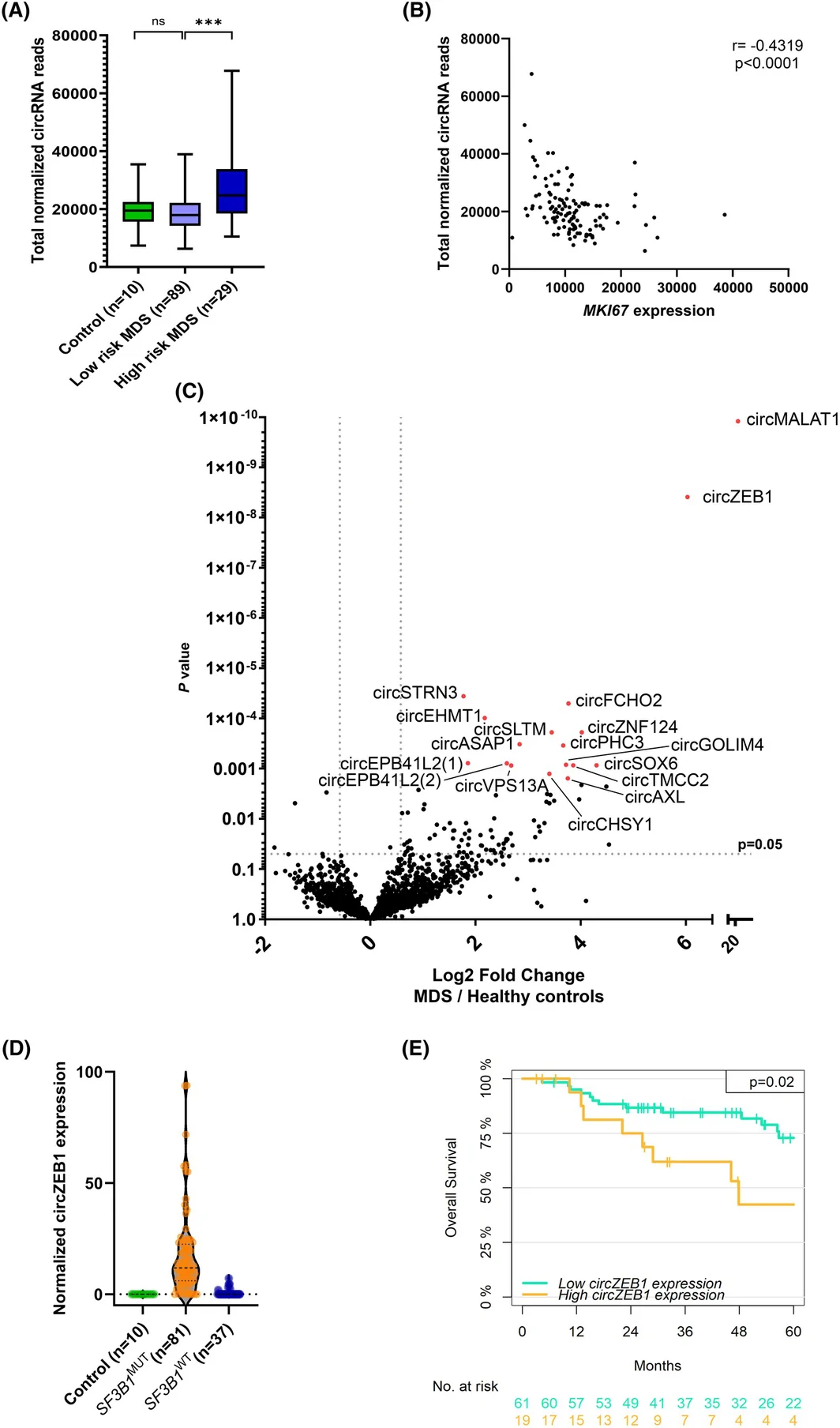
Circular RNA (circRNA) expression is also elevated in high risk myelodysplastic neoplasm (MDS) patients in the independent ring sideroblast cohort. (A) Total normalized circRNA reads by disease group, with MDS divided into Low Risk (International Prognostic Scoring System ‐Molecular (IPSS‐M) Very Low, Low, or Moderate Low risk groups; *n* = 89) and High Risk (IPSS‐M Moderate High, High or Very High risk groups; *n* = 29). Bars show median and quartiles, whiskers show range, comparison by Mann Whitney test (***P < 0.001, ns = not significant). (B) Total normalized circRNA reads plotted against *MKI67* expression, analysis by Spearman's correlation (*n* = 118). (C) Volcano plot depicting differential expression of circRNAs between patients with MDS (*n* = 118) and healthy controls (*n* = 10), including 2134 highly expressed circRNAs (cutoff determined by DESeq2 independent filtering). Raw *P* values are plotted, with significant p(adj) indicated by red dots. (D) Violin plot showing expression of circZEB1 in *SF3B1* ^MUT^ (*n* = 81) and *SF3B1* ^WT^ (*n* = 37) groups as well as healthy controls (*n* = 10), dotted lines show quartiles (median in bold). (E) Kaplan–Meier plot showing overall survival among patients with *SF3B1* mutations, divided by circZEB1 expression (High circZEB1 expression = top quartile).

### 3.9 circZEB1 is upregulated in the presence of *SF3B1* mutations and is associated with poorer survival

circRNA abundance was not significantly associated with overall survival in the RS cohort as a whole, but was associated with overall survival among patients with *SF3B1* mutations (*n* = 80, *P* = 0.02). However, this was not independent of the IPSS‐M (IPSS‐M *P* = 0.00005, total normalized circRNA reads *P* = 0.899). Differential expression analysis of MDS versus healthy controls revealed a clear skew toward upregulation (Fig. 5C). circZEB1 was among the most upregulated in MDS, again driven by high expression in patients with *SF3B1* mutations (Fig. 5D). Among patients with *SF3B1* mutations, high levels of circZEB1 (top quartile) were associated with poorer overall survival at 5 years (*P* = 0.02, Fig. 5E). This was not independent of the IPSS‐M (circZEB1 top quartile *P* = 0.2, IPSS‐M *P* = 0.0001). The incidence of AML was too small in this low‐risk cohort to enable the analysis of progression risk.

## 4 Discussion

In this study, ultra‐deep total RNA‐seq from a large, well‐characterized MDS patient cohort and age‐matched healthy controls led to several novel findings. Associations between circRNA abundance and disease presence, severity, and progression suggest aberrant expression is linked to disease biology. Importantly, the upregulation we have described is largely independent of changes in cognate linear gene expression. This suggests that there are other factors underlying the increase in circRNA abundance.

The association between circRNA expression and disease progression was observed at the level of total circRNA abundance but was strongest in a number of highly correlated MDS‐associated circRNAs. CMML showed less change in circRNA expression than MDS, relative to healthy controls, which may relate to different clonal architectures in the two diseases [34], or differences between dysplastic and proliferative phenotypes of CMML. In line with this, we found that the highest circRNA levels in the CMML group were found in cases with dysplastic CMML (data not shown).

Spliceosome mutations appear to be associated with differences in circRNA expression. There was a tendency for lower circRNA levels in the context of *SF3B1* mutations, with the exception of circZEB1. circRNA abundance tended to be higher in the presence of *U2AF1* or *SRSF2* mutations than *SF3B1*, though analysis was limited by the scarcity of spliceosome‐wild‐type patients and comutation complexity. While most patients with *SRSF2* mutations and high circRNA abundance also had *IDH2* mutations, no obvious comutation was associated with high circRNA expression in the case of *U2AF1*. Cluster analysis showed gene‐ and variant‐related patterns, indicating locus‐specific effects associated with perturbation of the spliceosome. One circRNA, circZEB1, was specifically upregulated in cases with *SF3B1* mutations in both cohorts and has also been reported previously [32]. Interestingly, silencing of *SF3B1* does not lead to increases in circZEB1, confirming that the circZEB1 expression changes are attributable to the mutant SF3B1 protein [32] and presumably alterations in its splicing behavior.

Cancer states are usually associated with a predominant downregulation of circRNAs linked to increased cellular proliferation [30, 35, 36, 37, 38], termed the circRNA dilution hypothesis [26]. Consistent with this, and the previous finding of Ki67^low^/BCL2^high^ cell populations in some patients with MDS [13], we have demonstrated an association between reduced cell turnover and increased circRNA abundance in MDS. However, proliferation does not appear to fully explain the changes in circRNA expression we have observed. Indeed, when examining correlations between individual circRNAs and selected genes, we found that over one‐third of the circRNAs, which were upregulated in MDS showed no correlation with *MKI67*, *PCNA*, or *BCL2*. Furthermore, the *SF3B1*‐associated elevation of circZEB1 occurred despite higher proliferation in these patients. Notably, correlations with innate immunity genes and cell turnover markers tended to co‐occur for the same circRNAs, while others displayed no correlation with either group of genes. This suggests that these two associations are not alternatives to one another, but rather have a shared relationship with circRNA expression, the exact nature of which cannot be elucidated here.

Assessing the functional impact of the circRNA upregulation that we observed is beyond the scope of this article and would be challenging for several reasons. First, there is no true MDS cell line, and cell line experiments lack recapitulation of the many interactions within the bone marrow microenvironment. Second, shared mechanisms may lead to biological consequences from a widespread upregulation of circRNAs, which cannot be mimicked experimentally.

## 5 Conclusions

This explorative study describes several features of circRNAs as an additional level of transcriptional complexity in MDS. High circRNA abundance is associated with high‐risk MDS and progression to AML. The alterations in circRNA expression may be partially attributable to reduced cellular proliferation and the influence of spliceosome mutations. Together, our findings support further investigation into the biological mechanisms and biomarker potential of circRNA in MDS.

## Conflict of interest

The authors declare no conflict of interest.

## Author contributions

EW, KG, JWH, MD, and LSK conceived of the idea for the project and planned the project. EW was involved in laboratory work, statistical analysis, data interpretation, and manuscript writing. MT was involved in data interpretation and analysis, in addition to variant filtering. GT and PM generated the data from the RS cohort and were involved in analysis planning and data interpretation; additionally, MC was involved in characterization of this cohort. CC planned and carried out FACS sorting. JSJ and BS carried out NGS panel sequencing and variant filtering; JSJ was additionally involved in RNA lab work. CS and KRJ were involved in patient inclusion and characterization. KW, BP, and JW were involved in strategy and initiation under the Danish Research Center for Precision Medicine in Blood Cancers, along with development of the manuscript. EHL contributed data from the RS cohort and was involved in analysis planning and data interpretation. KG and LSK were involved in project planning, data interpretation, and manuscript development. All authors contributed intellectual input and revised and approved the final manuscript.

## Supporting information


**Table S1.** Included genes in targeted next generation sequencing.
**Table S2.** FACS antibodies.
**Fig. S1.** Circular RNA (circRNA) abundance and expression patterns are altered in myelodysplastic neoplasms (MDS), cloncal cytopenia of uncertain significance (CCUS) and chronic myelomonocytic leukemia (CMML).
**Fig. S2.** Downregulated genes in the high circRNA abundance group.
**Fig. S3.** Correlation patterns are seen between circRNA expression and genes of interest.
**Fig. S4.** circRNA upregulation, independent to cognate linear gene expression, can also be seen in CCUS and CMML.
**Fig. S5.** circRNAs associated with risk of leukemic progression after exclusion of MDS‐SF3B1.

## Data Availability

The raw RNA sequencing data from patients and healthy controls generated in this study are available under restricted access, since all genomic data are considered sensitive personal data according to Danish Law and the European Union General Data Protection Regulation (GDPR), and thus cannot be shared with third parties without prior approval. To access the RNA data, an application must be sent to kirsten.groenbaek@regionh.dk. Applications will be reviewed by the DCCC/PTH board and subsequently by the Danish Data Protection Agency (DDPA). Access can only be granted for research purposes, and only if a data processor or data transfer agreement can be made in accordance with Danish and European law at the given time. Deidentified individual participant data from the Swedish RS cohort are available in the Swedish National Data Service repository at https://doi.org/10.48723/zt59‐8x04 upon request.
