# Supplementary material for: Circular RNA expression landscapes in myelodysplastic neoplasms: Associations with mutational signatures and disease progression
Source: Mol Oncol. 2026 Feb 19:10.1002/1878-0261.70208. Online ahead of print. doi: 10.1002/1878-0261.70208 (PMC13399211; doi:10.1002/1878-0261.70208)
Supplement: Supplementary file 1 — Table S1. Included genes in targeted next generation sequencing. Table S2. FACS antibodies. Fig. S1. Circular RNA (circRNA) abundance and expression patterns are altered in myelodysplastic neoplasms (MDS), cloncal cytopenia of uncertain significance (CCUS) and chronic myelomonocytic leukemia (CMML). Fig. S2. Downregulated genes in the high circRNA abundance group. Fig. S3. Correlation patterns are seen between circRNA expression and genes of interest. Fig. S4. circRNA upregulation, independent to cognate linear gene expression, can also be seen in CCUS and CMML. Fig. S5. circRNAs associated with risk of leukemic progression after exclusion of MDS‐SF3B1. [file MOL2-9999-0-s001.pdf]

Supplementary Figures and Tables

Table S1: Included genes in targeted next generation sequencing

Gene	Preferred transcript
**ASXL1**	ENST00000375687.10
**BCOR**	ENST00000378444.9
**CALR**	ENST00000316448.10
**CBL**	ENST00000264033.6
**CEBPA**	ENST00000498907.3
**DNMT3A**	ENST00000264709.7
**ETNK1**	ENST00000671733.1
**ETV6**	ENST00000396373.9
**EZH2**	ENST00000320356.7
**FLT3**	ENST00000241453.12
**GATA2**	ENST00000341105.7
**GNB1**	ENST00000378609.9
**IDH1**	ENST00000415913.5
**IDH2**	ENST00000330062.8
**JAK2**	ENST00000381652.4
**KIT**	ENST00000288135.6
**KMT2A**	ENST00000534358.8
**KRAS**	ENST00000311936.8
**NPM1**	ENST00000296930.10
**NRAS**	ENST00000369535.5
**PHF6**	ENST00000370803.8
**PPM1D**	ENST00000305921.8
**PRPF8**	ENST00000304992.11
**PTPN11**	ENST00000351677.7
**RUNX1**	ENST00000675419.1
**SETBP1**	ENST00000649279.2
**SF3B1**	ENST00000335508.11
**SRSF2**	ENST00000359995.10
**STAG2**	ENST00000371145.8
**TET2**	ENST00000380013.9
**TP53**	ENST00000269305.9
**U2AF1**	ENST00000291552.9
**WT1**	ENST00000452863.10
**ZRSR2**	ENST00000307771.8

Table S2: FACS antibodies

Antibody	Clone	Vendor	Catalog reference
**CD3 APC-Cy7**	SK7	BD	557832
**CD19 BV786**	HIB19	BD	740968
**CD34 BV605**	8G12	BD	745247
**CD45 V450**	HI30	BD	560367

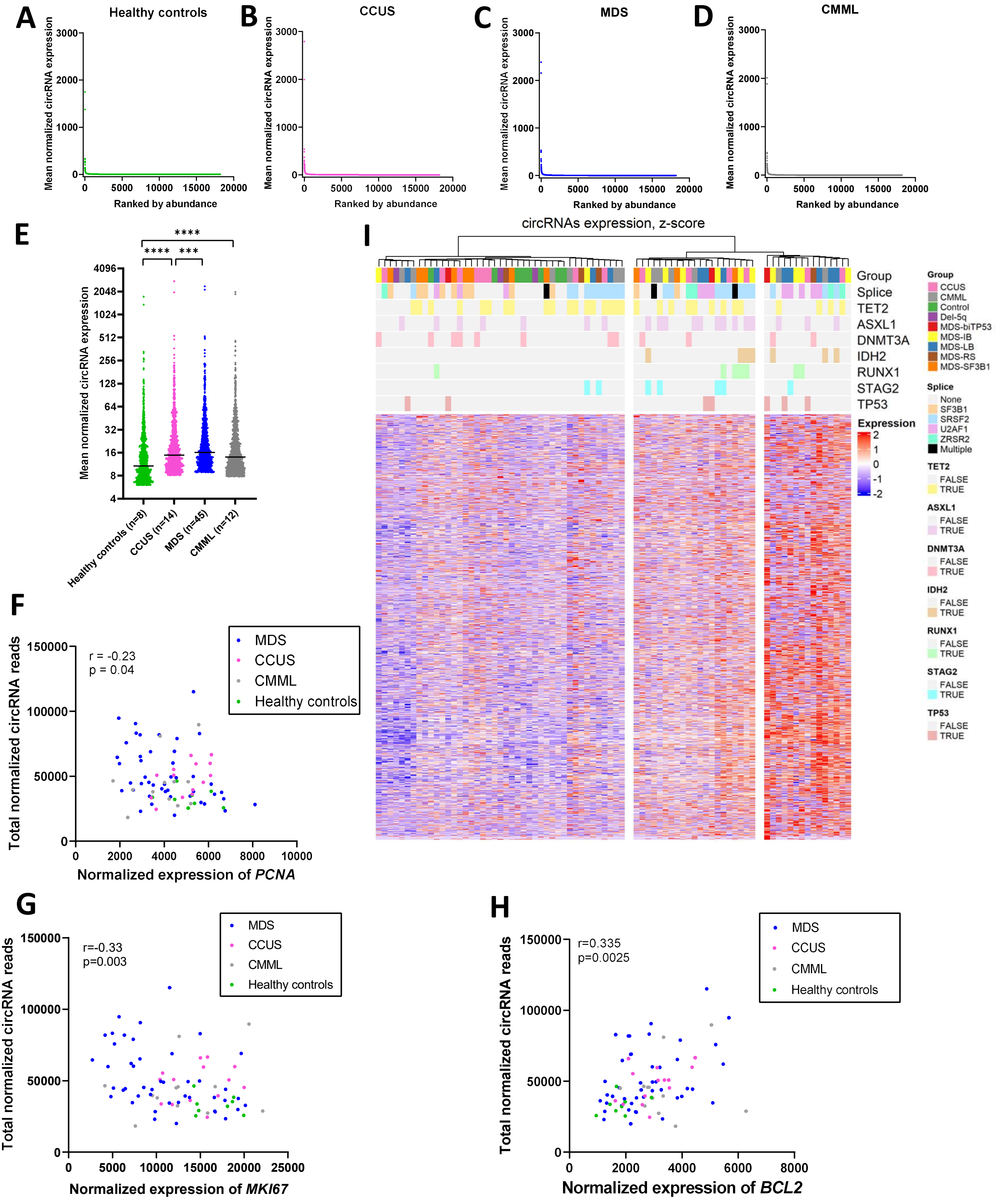

Supplementary figure 1: Circular RNA (circRNA) abundance and expression patterns are altered in myelodysplastic neoplasms (MDS), cloncal cytopenia of uncertain significance (CCUS) and chronic myelomonocytic leukemia (CMML). A-D) Groupwise mean normalized reads for the 18,235 detected circRNAs, plotted in order of abundance in healthy controls (A), CCUS (B), MDS (C) and CMML (D). **E)** Mean circRNA expression levels of the 1000 most abundant circRNAs for each group, comparison by Mann-Whitney tests, *** indicates p value <0.001, **** indicates p value <0.0001. **F)** Unsupervised clustering of all circRNAs with a mean expression ≥5 in at least one group (n=3300) using Euclidean clustering method. **G-I)** Scatter plots of total normalized cirRNA reads against expression of the genes PCNA (G), MKI67 (H) and BCL2 (I), groups indicated by colour.

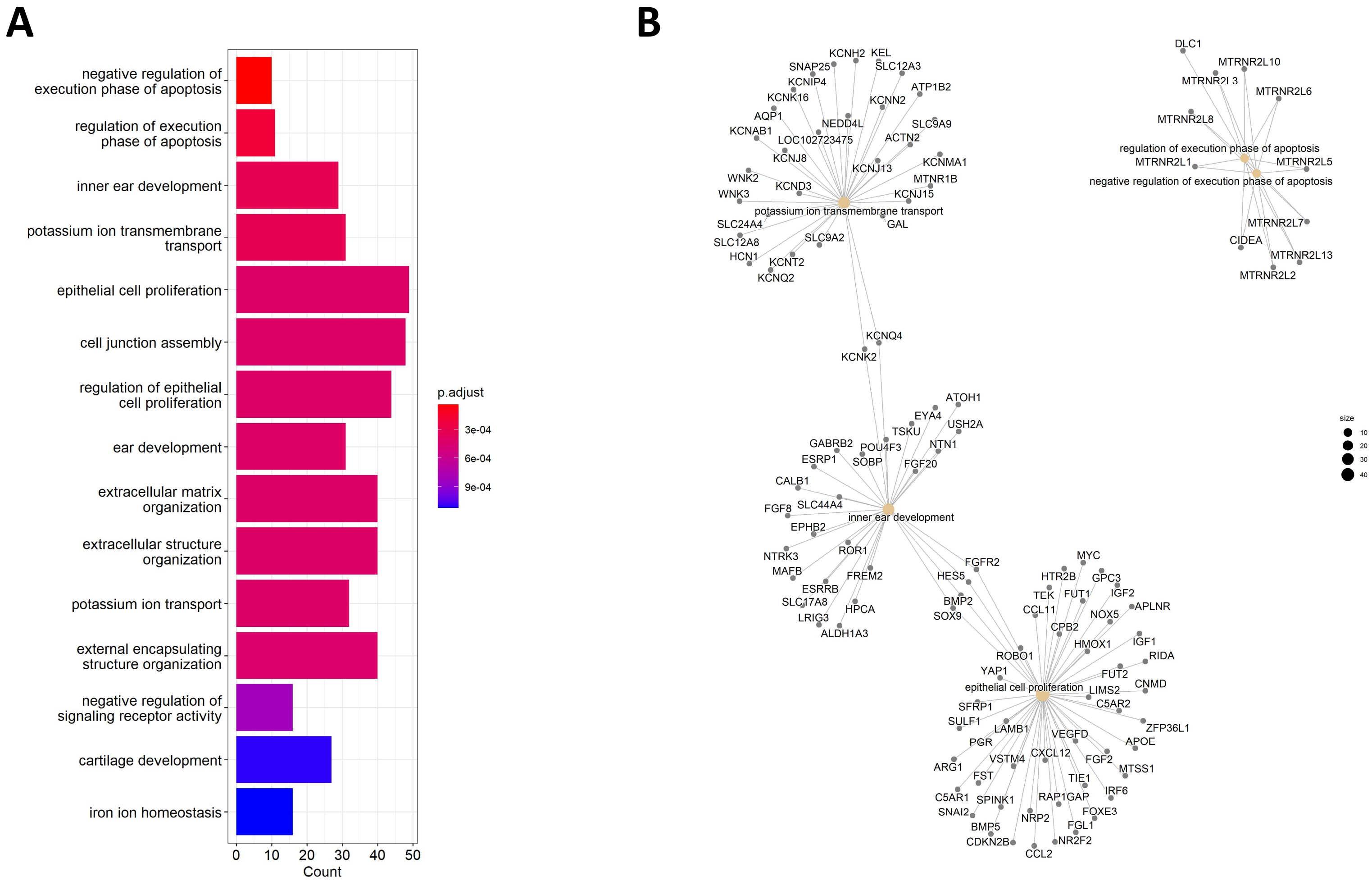

Supplementary figure 2. Downregulated genes in the high circRNA abundance group. A) GO analysis of genes downregulated in patients with high circRNA abundance, showing top 15 terms. **B**) Cnet plot showing the downregulated genes associated with the top 5 terms from panel C.

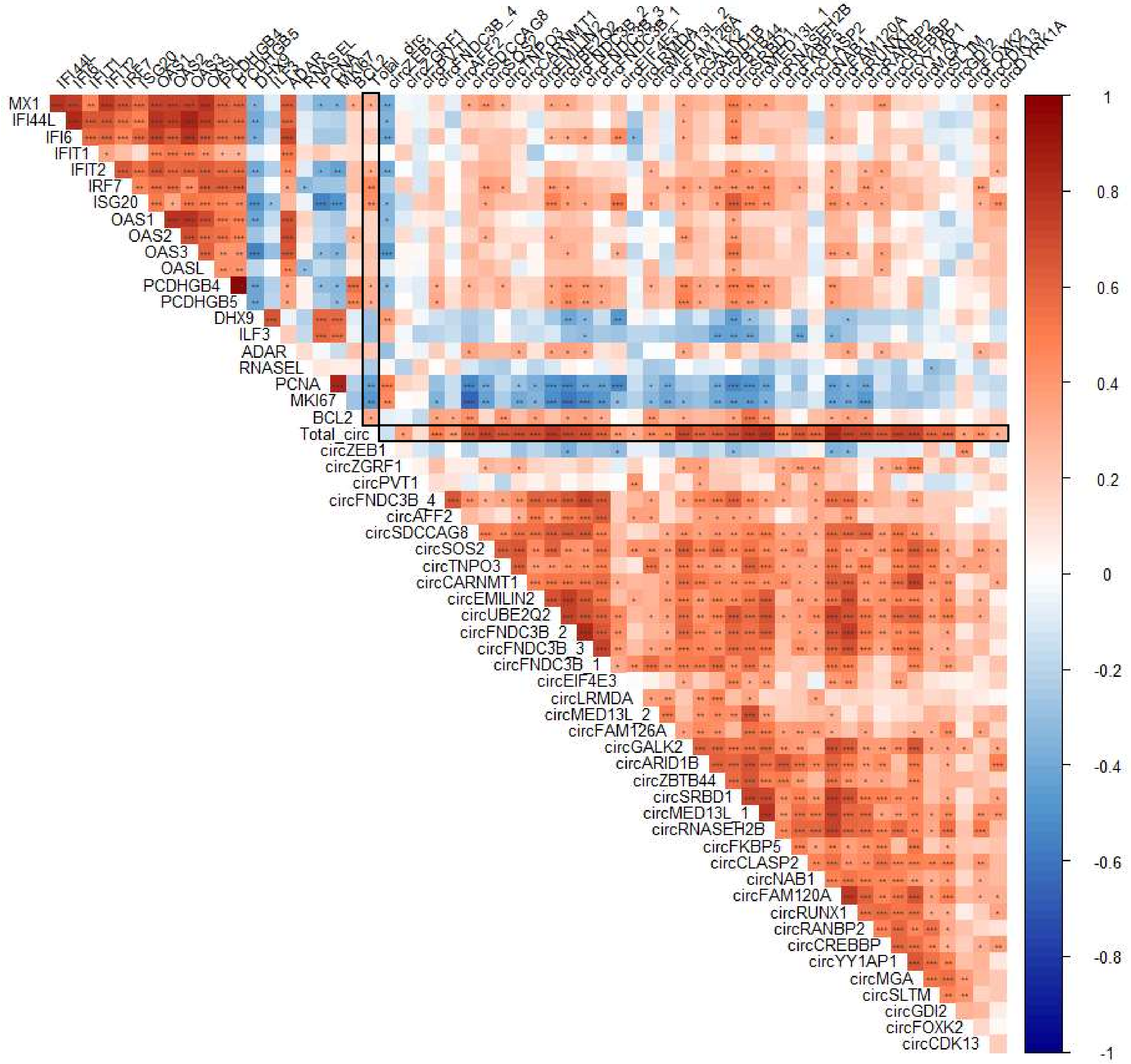

Supplementary figure 3. Correlation patterns are seen between circRNA expression and genes of interest. Correlation plot including selected genes involved in immune pathways, RNA binding, proliferation, and apoptosis, along with total circRNA abundance (marked with black box) and expression of the 38 circRNAs which are upregulated in MDS relative to healthy controls. This analysis including the 45 patients with MDS.

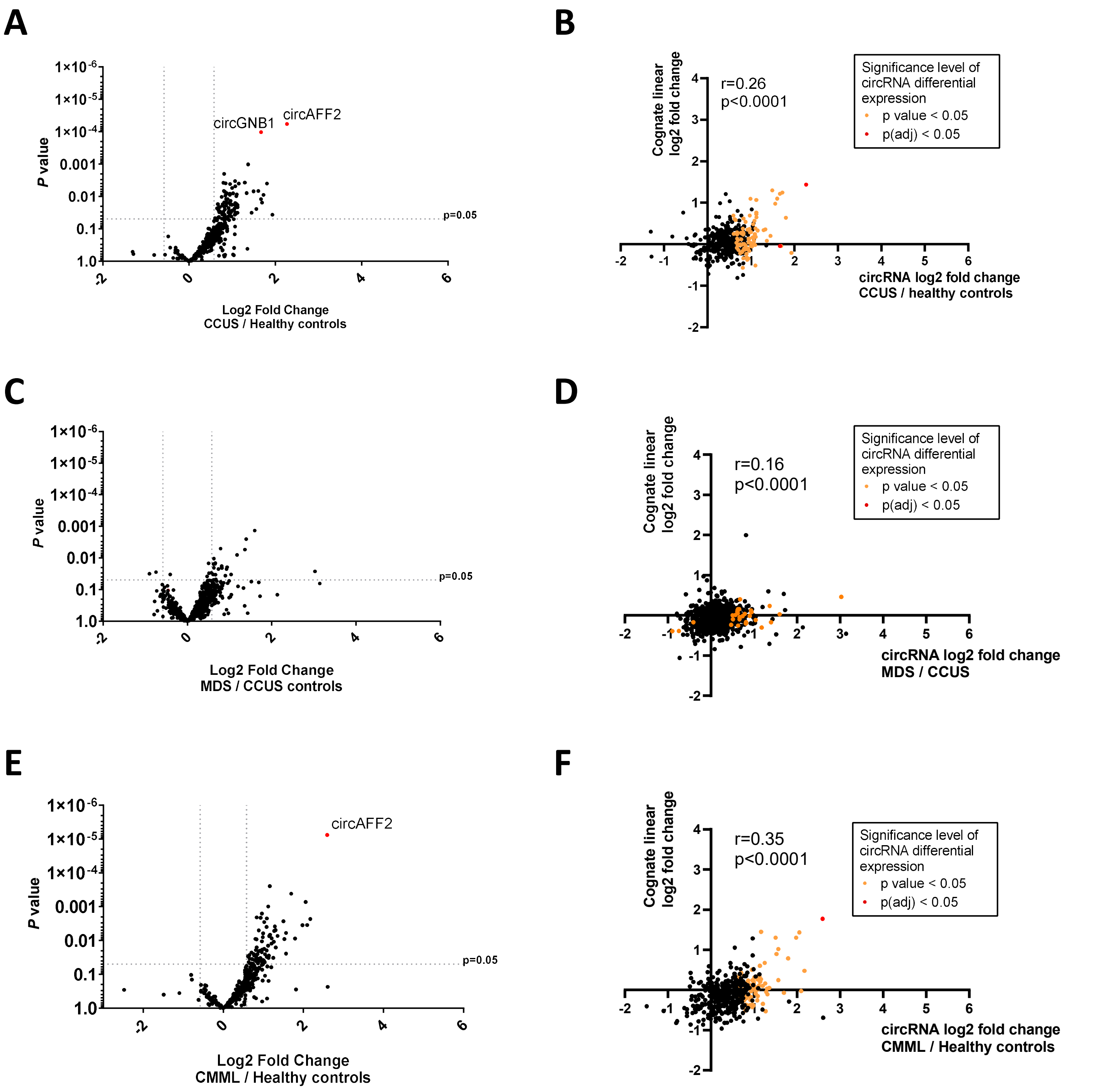

Supplementary figure 4. circRNA upregulation, independent to cognate linear gene expression, can also be seen in CCUS and CMML. A) Volcano plot of differential expression of circRNAs for patients with CCUS vs. healthy controls, including 431 highly expressed circRNAs (cutoff determined by DESeq2 independent filtering). Raw p value is plotted, whilst red dots indicate significant p(adj). **B)** Log2 fold change for circRNAs plotted against log2 fold change of linear mRNAs from the same locus, showing all circRNAs from panel A. Orange dots indicate circRNAs which are differentially expressed (sig. unadjusted p value) and red dots indicate significant p(adj), r and p by Spearman's correlation. **C)** Volcano plot of differential expression of circRNAs for MDS vs. CCUS, including the 1000 most abundant circRNAs (independent filtering not possible). Raw p value is plotted, whilst red dots indicate significant p(adj). **D)** Log2 fold change for circRNAs plotted against log2 fold change of linear mRNAs from the same locus, showing all circRNAs from panel C. Orange dots indicate circRNAs which are differentially expressed (sig. unadjusted p value) and red dots indicate significant p(adj), r and p by Spearman's correlation. **E)** Volcano plot of differential expression of circRNAs for CMML vs. Healthy controls, including 431 highly expressed circRNAs (cutoff determined by DESeq2 independent filtering). Raw p value is plotted, whilst red dots indicate significant p(adj). **F)** Log2 fold change for circRNAs plotted against log2 fold change of linear mRNAs from the same locus, showing all circRNAs from panel E. Orange dots indicate circRNAs which are differentially expressed (sig. unadjusted p value) and red dots indicate significant p(adj), r and p by Spearman's correlation.

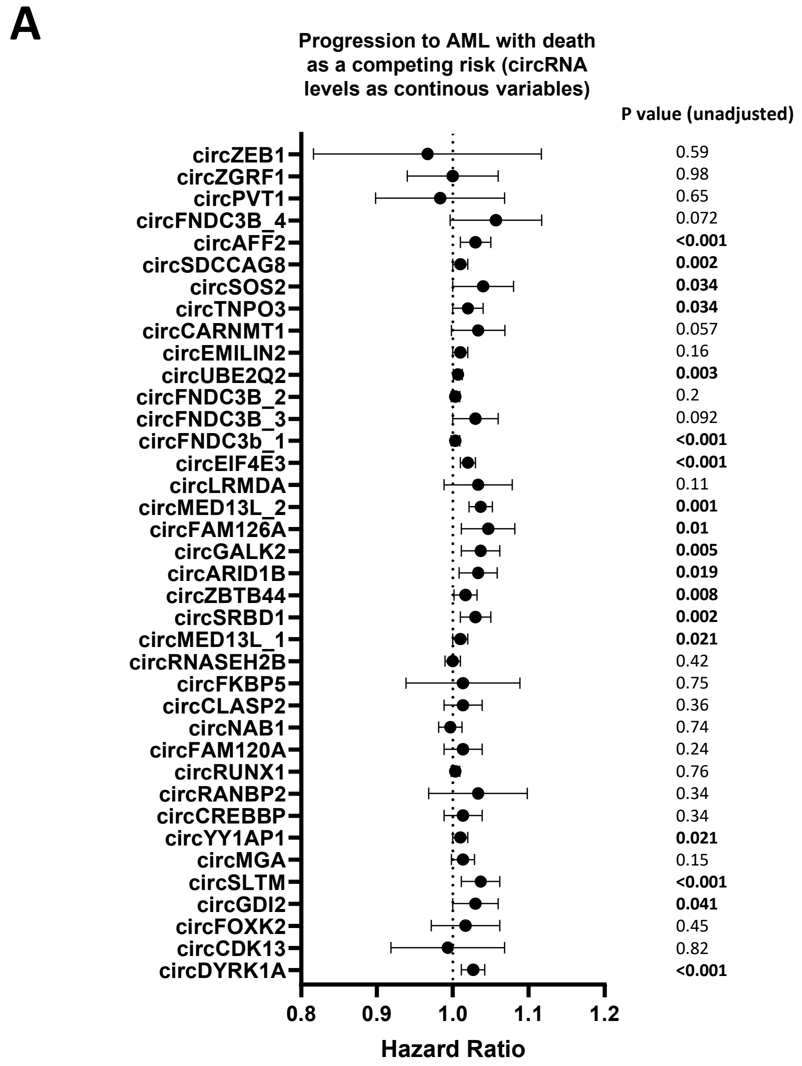

Supplementary figure 5. circRNAs associated with risk of leukemic progression after exclusion of MDS-SF3B1. Forest plot showing the results of univariate analyses for risk of progression to acute myeloid leukemia (AML) with death as a competing risk for patients with MDS excluding the MDS-SF3B1 group, for each of the 38 circRNAs which were upregulated in MDS relative to healthy controls. Here, the circRNAs are assessed as continuous variables.
